# Nerandomilast is Associated With Altered Alveolar Epithelial Cell State Dynamics While Attenuating Fibrotic Remodeling in Pulmonary Fibrosis

**DOI:** 10.1111/gtc.70149

**Published:** 2026-10-03

**Authors:** Nanako Hamada, Yoshiaki Hayashi, Daisuke Motooka, Atsushi Kuwahara, Shizuo Akira, Kiyoharu Fukushima

**Affiliations:** ^1^ Laboratory of Host Defense World Premier Institute Immunology Frontier Research Center (WPI‐IFReC), The University of Osaka Suita Japan; ^2^ Department of Host Defense Research Institute for Microbial Diseases (RIMD), The University of Osaka Suita Japan; ^3^ NGS Core Facility Research Institute for Microbial Diseases (RIMD), The University of Osaka Suita Japan; ^4^ Integrated Frontier Research for Medical Science Division Institute for Open and Transdisciplinary Research Initiatives, The University of Osaka Suita Japan; ^5^ Center for Advanced Modalities and DDS (CAMaD) The University of Osaka Suita Japan; ^6^ Bioinformatics Center Research Institute for Microbial Diseases (RIMD), The University of Osaka Suita Japan; ^7^ Department of Respiratory Medicine and Clinical Immunology The University of Osaka Graduate School of Medicine Suita Japan

## Abstract

Pulmonary fibrosis is a progressive and life‐threatening lung disorder characterized by excessive fibrogenesis and impaired respiratory function. Nerandomilast, a preferential phosphodiesterase 4B inhibitor, is currently approved for the treatment of idiopathic pulmonary fibrosis and progressive pulmonary fibrosis in several countries following its demonstrated clinical efficacy in Phase III trials. However, its cellular mechanisms of action remain incompletely understood. In this study, we investigated the effects of nerandomilast on a bleomycin‐induced mouse model of lung fibrosis. Nerandomilast treatment attenuates fibrotic remodeling and preserves alveolar epithelial architecture. Single‐cell transcriptomic analyses revealed altered alveolar epithelial cell state dynamics accompanied by increased cell cycle‐associated pathways in alveolar type 2 cells and an increased relative abundance of alveolar type 2 cells. Consistent with the single‐cell findings, nerandomilast was associated with an increased SFTPC‐positive area and the preservation of alveolar epithelial architecture in vivo. Collectively, our findings suggest that nerandomilast attenuates fibrotic remodeling while modulating alveolar epithelial cell state dynamics, which may provide a possible cellular explanation for its clinical efficacy in patients with pulmonary fibrosis.

## Graphical abstract

Nerandomilast treatment is associated with altered AT2 cell transcriptional programs and alveolar epithelial cell state dynamics, along with attenuated fibrotic remodeling and preservation of alveolar epithelial architecture. These findings suggest that nerandomilast may modulate alveolar epithelial responses during lung injury and repair.

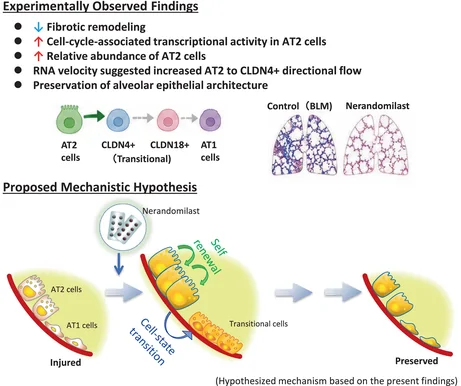

## 1 Introduction

Pulmonary fibrosis (PF) is a chronic and progressive lung disorder characterized by excessive fibrogenesis, declining respiratory function, and a complex multifactorial etiology (Wang et al. 2025; Fan et al. 2025). As a life‐threatening disease associated with poor clinical outcomes and substantial mortality, PF poses a major global health challenge (Cottin et al. 2019). Nevertheless, the pathogenic mechanisms driving the initiation and progression of PF are not fully understood.

PF results from the impaired regeneration of the alveolar epithelium after injury, and recent studies have highlighted alveolar epithelial cells as key drivers of disease development (Wang et al. 2023; Katzen and Beers 2020). Repetitive damage to epithelial cells causes them to lose their ability to repair, leading to destruction of the alveolar structure and triggering fibrotic responses (Moss et al. 2022). Thus, alveolar epithelial cells are intimately involved in the progression of pulmonary fibrosis, and their targeted regulation represents a promising therapeutic strategy.

Phosphodiesterase 4 (PDE4) inhibitors exert anti‐inflammatory and anti‐fibrotic effects by suppressing cytokine production and limiting immune cell activation through increased intracellular cyclic adenosine monophosphate (cAMP) expression levels (Blauvelt et al. 2023; Aringer et al. 2024). Several pan‐PDE4 inhibitors, such as roflumilast for COPD (Cilli et al. 2019), apremilast for psoriatic arthritis (Kavanaugh et al. 2019), and crisaborole for atopic dermatitis (McDowell and Olin 2019), have already been approved for clinical use. However, pan‐PDE4 inhibitors, when administered systemically, are associated with adverse gastrointestinal events that often prevent patients from reaching therapeutic doses adequate for the treatment of progressive diseases, such as PF (Aringer et al. 2024; Fan et al. 2024). Nerandomilast, a preferential phosphodiesterase 4B (PDE4B) inhibitor, is a novel and promising drug for PF with a nine‐fold preference for the PDE4B versus PDE4D isoenzyme (Herrmann et al. 2022). This unique selection results in a decrease in adverse effects, such as nausea and emesis, largely due to reduced pan‐PDE4 inhibition in non‐target tissues (Li et al. 2018; Richeldi et al. 2022). In a Phase III trial, treatment with nerandomilast resulted in a smaller decline in forced vital capacity (FVC) compared to placebo over a 52‐week period (Richeldi et al. 2025). Based on these clinical findings, nerandomilast has been approved for the treatment of idiopathic pulmonary fibrosis (IPF) and progressive pulmonary fibrosis in several countries. These approvals established nerandomilast as a breakthrough therapeutic agent. Despite these remarkable clinical results, the precise cellular effects of nerandomilast on lung tissue dynamics remain unclear. While PDE4B inhibition has mainly been investigated in the context of inflammatory responses in immune cells, its relationship with alveolar epithelial dynamics during lung repair remains unclear. Hence, we explored the cellular responses induced by nerandomilast in the lung to better understand the mechanisms underlying its anti‐fibrotic effects.

In this study, we performed single‐cell RNA sequencing (scRNA‐seq) along with molecular and histological analyses. We found that nerandomilast treatment attenuated fibrotic remodeling and was associated with preservation of alveolar epithelial architecture in a bleomycin (BLM)‐induced mouse model of lung fibrosis. Furthermore, scRNA‐seq analyses revealed the modulation of alveolar epithelial cell‐state dynamics, including increased cell‐cycle‐associated transcriptional activity and directional flow from alveolar type 2 (AT2) cells toward transitional epithelial states following nerandomilast treatment. These transcriptomic findings were consistent with the histological evidence of an increased SFTPC‐positive epithelial area and preservation of the alveolar epithelial architecture.

Collectively, our findings provide new insights into the cellular changes associated with nerandomilast treatment in experimental pulmonary fibrosis and suggest that nerandomilast modulates alveolar epithelial cell‐state dynamics.

## 2 Results

### 2.1 Nerandomilast Significantly Ameliorates BLM‐Induced Lung Fibrosis

We evaluated the therapeutic effects of nerandomilast using mouse models of BLM‐induced lung fibrosis. Nerandomilast was orally administered twice daily, starting on Day 8 after BLM administration. Samples were harvested on Days 10 and 14 (Figure 1A). Body weight loss on Day 14 was significantly attenuated in nerandomilast‐treated mice relative to that for the controls (two‐tailed Mann–Whitney *U* test, *p* = 0.0099; Figure 1B). Furthermore, gross images of the lungs and staining with hematoxylin and eosin (HE) and Masson's trichrome demonstrated reduced alveolar structural disruption and collagen deposition following nerandomilast treatment (Figure 1C,D). Quantitative analyses further confirmed a significant reduction in collagen accumulation, as assessed by Masson's trichrome staining and hydroxyproline assay (*p* = 0.0064; Figure 1E, *p* = 0.0482; Figure 1F). In addition, qPCR analyses revealed a significantly decreased expression of the fibrotic markers *Col1a1* (*p* = 0.0436) and *Tgfb1* (*p* = 0.0024) in mice treated with nerandomilast (Figure 1G). Collectively, these findings indicated that nerandomilast attenuated BLM‐induced pulmonary fibrosis in mice.

**FIGURE 1:**
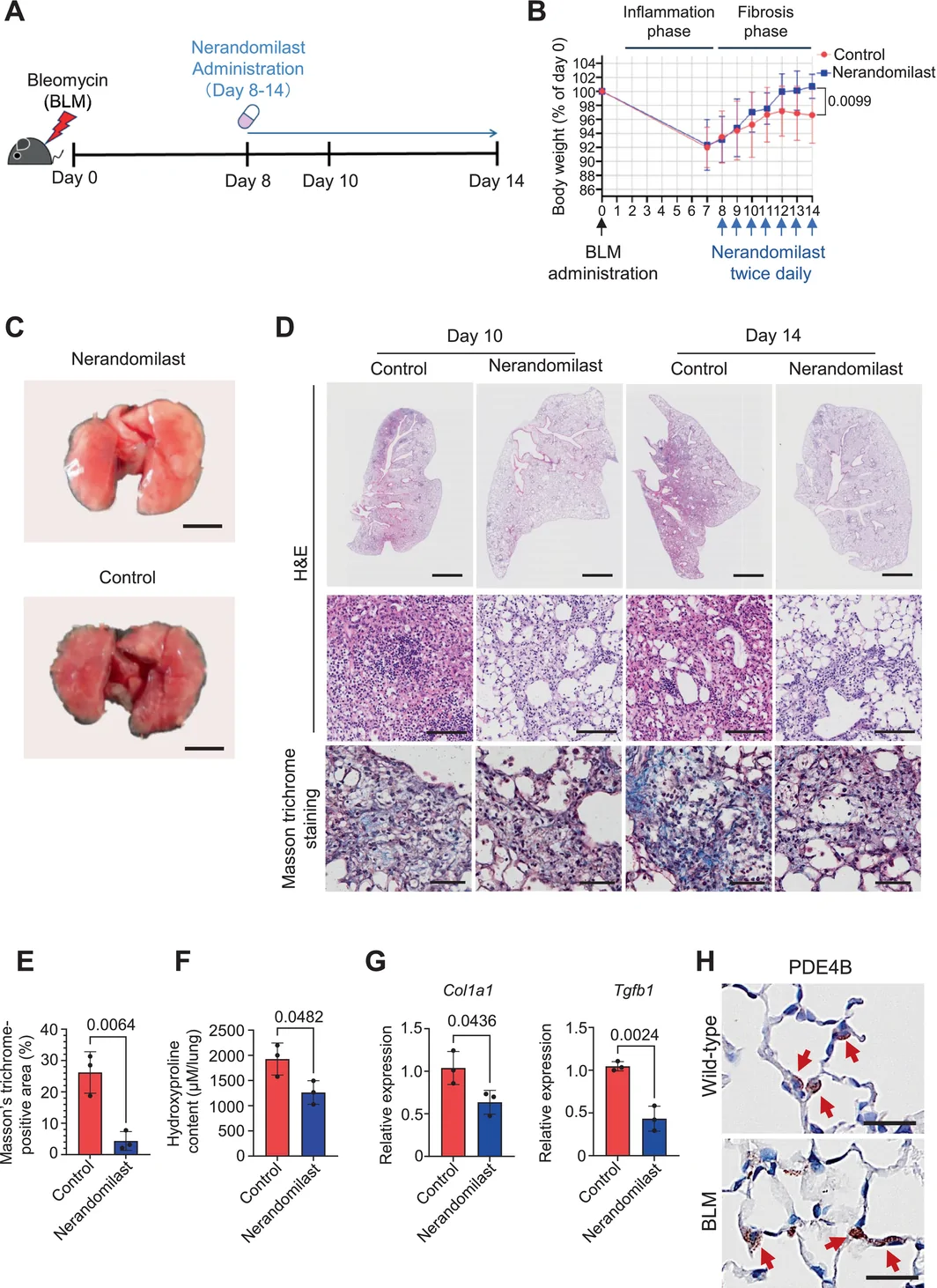
Nerandomilast ameliorates bleomycin‐induced lung fibrosis. (A) Experimental design and treatment schedule. (B) Body weight was monitored throughout the experimental period. Statistical comparison was performed between two groups on Day 14 using a two‐tailed Mann–Whitney *U* test. Data are presented as mean ± SD (*n* = 7 mice per group). (C) Representative gross images of lungs collected on Day 14. Scale bars: 5 mm. (D) Representative H&E and Masson's trichrome staining of 3 μm FFPE lung sections collected on Days 10 and 14. Scale bar: upper, 2 mm; middle, 100 μm; lower, 50 μm. (E) Quantification of the collagen‐positive area based on Masson's trichrome staining on Day 14. Each point represents one mouse (*n* = 3 mice per group). (F) Quantification of lung hydroxyproline concentrations on Day 14. Each point represents one mouse (*n* = 3 mice per group). (G) Quantitative PCR analysis of fibrosis‐related genes in lung tissues collected on Day 14. Relative mRNA expression levels of *Col1a1* and *Tgfb1* were normalized using the ΔΔCt method. Data are presented as mean ± SD. Each point represents one mouse (*n* = 3 mice per group). *p* < 0.05 by student's *t*‐test. (H) Representative immunostaining of PDE4B in lungs from untreated and BLM‐treated mice on Day 10. Scale bar: 25 μm.

To elucidate the tissue distribution of its primary target, PDE4B, immunohistochemical analysis was performed. The results revealed that the morphological features of PDE4B‐positive cells were consistent with those of AT2 cells in the alveolar regions of mice with and without BLM‐induced lung fibrosis (Figures 1H and S1A,B).

### 2.2 Single‐Cell Transcriptomic Profiling Identifies Distinct Alveolar Epithelial Cell States

To further explore the effects of nerandomilast on the alveolar epithelium, we performed scRNA‐seq of lungs from mice administered BLM with or without nerandomilast treatment and subsequently analyzed the epithelial cell populations. Samples were collected at three time points after BLM administration (Days 8, 10, and 14), together with the uninjured control lung on Day 0 (Day 0: *n* = 1; others: *n* = 2 biological replicates; Figure 2A). Single‐cell transcriptional profiles were visualized in two dimensions using the uniform manifold approximation and projection (UMAP) method (Figure 2B). A total of 252,113 lung cells were divided into 11 cell types that were manually annotated using canonical marker genes (e.g., EPCAM for lung epithelial cells, CD68 for macrophages, and PECAM1 for endothelial cells) based on published single‐cell lung atlases (Figures 2C and S2A) (Negretti et al. 2021; Curras‐Alonso et al. 2023). Sub‐cluster analysis of the alveolar epithelial population identified four distinct epithelial cell states: alveolar type 1 (AT1), AT2, CLDN4^+^, and CLDN18^+^ (Figure 2D). AT1 and AT2 identities were defined by the expression of canonical markers, including *Ager* and *Emp2* for AT1 cells, and *Sftpc* and *Lamp3* for AT2 cells (Figure 2E). The remaining two clusters, CLDN4^+^ and CLDN18^+^, had high expressions of transitional epithelial markers, such as *Krt8* with relatively low expression of AT1 and AT2 markers, and were classified as transitional epithelial states (Figure 2E,F). Differential expression analysis of these two transitional clusters revealed that they were characterized by enrichment of *Cldn4*, *Gclc*, *Malat1*, and *Sfn*, whereas the other clusters exhibited elevated expression levels of *Cldn18*, *Prdx6*, *Clic3*, and *Clic5*. Accordingly, these clusters were designated CLDN4^+^ and CLDN18^+^ transitional cells (Figure 2F,G). Notably, the CLDN18^+^ population also exhibited a higher expression of AT1 markers (*Hopx*, *Ager*, and *Emp2*) than the CLDN4^+^ population, suggesting that CLDN18^+^ cells represent a more advanced transitional state poised for maturation into AT1 cells (Figure 2G).

**FIGURE 2:**
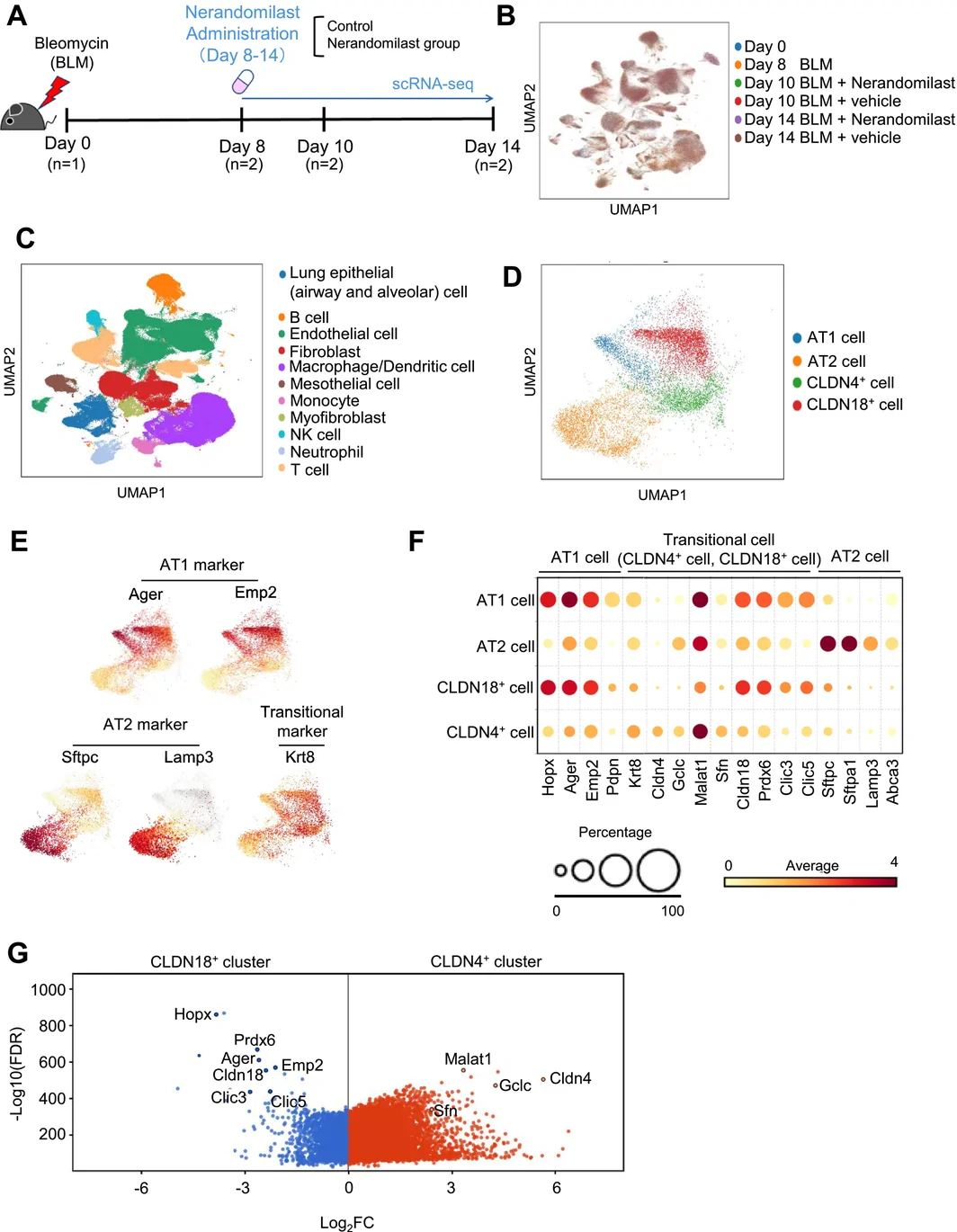
Single‐cell transcriptomic profiling identifies distinct alveolar epithelial cell states. (A) Experimental design and scRNA‐seq workflow. (B) UMAP visualization of all cells colored according to time point and treatment group. (C) UMAP visualization of major lung cell populations identified by scRNA‐seq. (D) UMAP visualization of alveolar epithelial cell subclusters. (E) UMAP visualization showing the expression of the indicated genes in epithelial populations. (F) Gene expression of key markers in each distinctive epithelial cluster. (G) Volcano plot showing differentially expressed genes enriched in CLDN4^+^ and CLDN18^+^ transitional epithelial cell populations identified using the Venice algorithm.

### 2.3 Nerandomilast Modulates AT2 Cell Transcriptional Programs and Alveolar Epithelial Cell State Dynamics

To gain mechanistic insights into the transcriptional programs in AT2 cells induced by nerandomilast, we analyzed differentially expressed genes in AT2 cells on Day 14 following BLM administration. Kyoto Encyclopedia of Genes and Genomes (KEGG) pathway enrichment analysis was performed using the top 100 upregulated and downregulated genes following nerandomilast treatment, ranked by the Wald statistic (Stat) (Table S1). This analysis revealed a significant downregulation of pathways related to efferocytosis, complement and coagulation cascades, and ECM‐receptor interactions following nerandomilast treatment (Figure 3A). In addition, several disease‐associated pathways sharing inflammatory signaling components, including COVID‐19 and rheumatoid arthritis, were reduced (Figure 3A), suggesting attenuation of injury‐ and remodeling‐associated transcriptional programs in AT2 cells.

**FIGURE 3:**
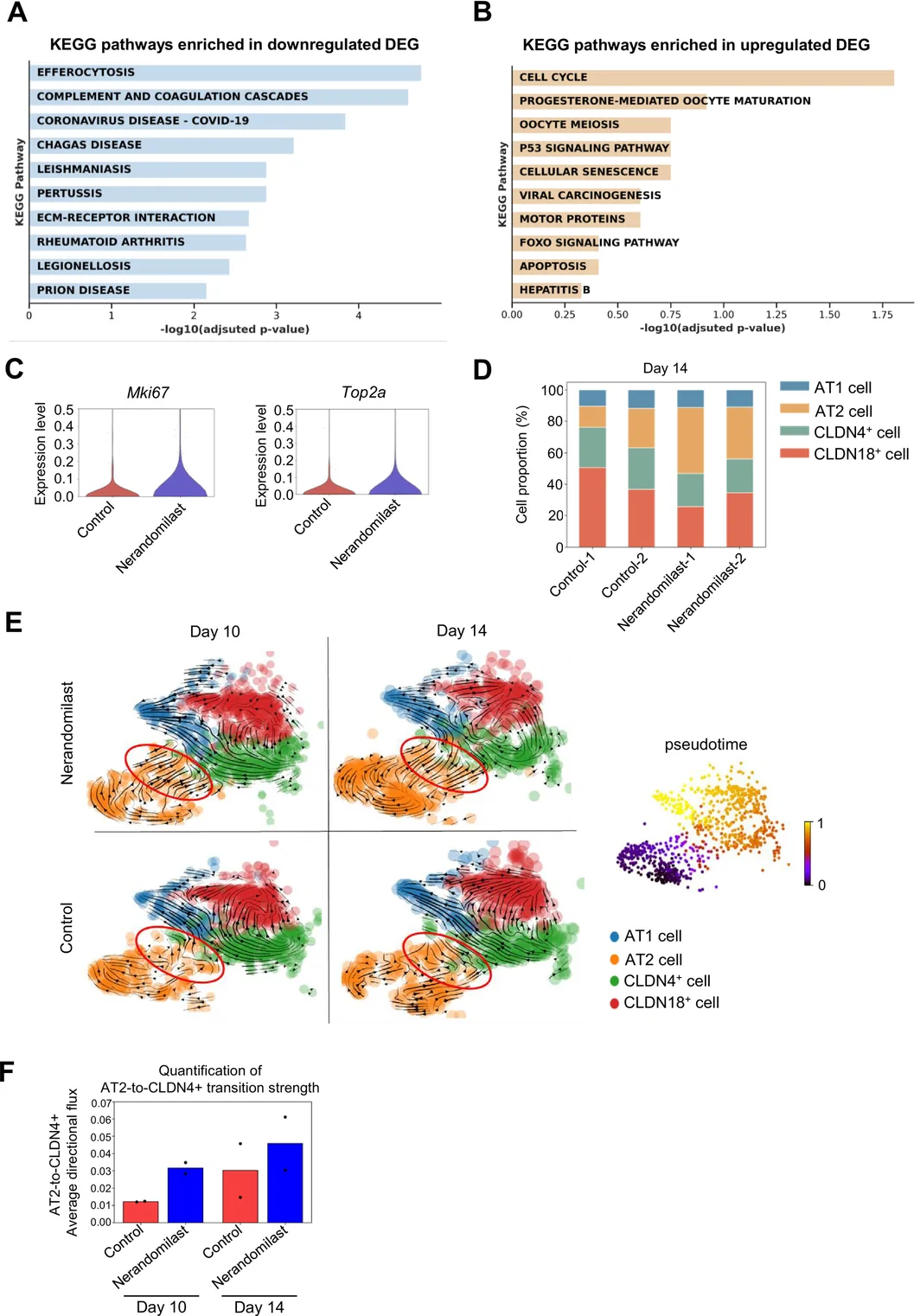
Nerandomilast is associated with altered AT2 cell states and epithelial cell‐state dynamics. (A) Top 10 KEGG pathways enriched among the top 100 downregulated differentially expressed genes (DEGs) ranked by the Wald statistic (stat) in AT2 cells from nerandomilast‐treated mice compared with controls on Day 14 after BLM administration. Pathway significance was assessed using Benjamini–Hochberg‐adjusted *p* values. (B) Top 10 KEGG pathways enriched among the top 100 upregulated DEGs ranked by the Wald statistic (stat) in AT2 cells from nerandomilast‐treated mice compared with controls on Day 14 after BLM administration. Pathway significance was assessed using Benjamini–Hochberg‐adjusted *p* values. (C) Violin plots showing the expression of cell cycle‐associated genes (*Mki67* and *Top2a*) in AT2 cells on Day 14. (D) Relative abundance of alveolar epithelial cell populations (AT1, AT2, CLDN4^+^, and CLDN18^+^) on Day 14. (*n* = 2 biological replicates per group). (E) RNA velocity and RNA velocity‐derived pseudotime analysis of alveolar epithelial cell states. Pseudotime trajectories were inferred using RNA velocity analysis with AT2 cells defined as the root state. A representative trajectory from a single mouse in the nerandomilast‐treated group on Day 14 is shown. (F) Quantification of RNA velocity directionality along the AT2‐to‐CLDN4^+^ trajectory. Directionality was calculated as the average projection of RNA velocity vectors onto the reference direction within the predefined region of interest. Each dot represents one biological replicate (*n* = 2 per group).

In contrast, cell cycle was the most significantly upregulated pathway in the nerandomilast‐treated group (Figure 3B). Consistent with this finding, the expression levels of the cell‐cycle‐associated markers *Mki67* (encoding Ki67) and *Top2a* (encoding Topoisomerase IIα) were increased in AT2 cells following nerandomilast treatment (Figure 3C). To provide a comprehensive view of alveolar epithelial cell composition, we quantified their relative abundance. No obvious differences in the epithelial cell composition were observed between the control and nerandomilast‐treated group on Day 10 (Figure S2B). In contrast, on Day 14, the nerandomilast‐treated group exhibited an increased relative abundance of AT2 cells and a reduced proportion of transitional cells, whereas the proportion of AT1 cells remained largely unchanged (Figure 3D). Collectively, these findings suggest that nerandomilast attenuates injury‐ and remodeling‐associated transcriptional programs in AT2 cells while promoting cell cycle‐associated transcriptional activity and increasing the relative abundance of AT2 cells.

To investigate the dynamics of AT2 cells, we performed RNA velocity analysis. The velocity vector fields indicated directional flow from AT2 cells toward the CLDN4^+^ cell state (Figure 3E). Notably, this directional flow was more pronounced in the nerandomilast‐treated mice than in the control mice on Days 10 and 14 (Figure 3E). Quantitative analysis suggested a trend toward greater AT2‐to‐CLDN4^+^ transition strength following nerandomilast treatment (Figure 3F). Pseudotime was inferred based on RNA velocity analysis. The results of the RNA velocity‐derived pseudotime distribution were consistent with the transcriptional relationships identified by the sub‐clustering analysis and the inferred transcriptional progression from AT2 cells through CLDN4^+^ and CLDN18^+^ transitional states toward the AT1 lineage (Figure 3E). Together with the increased relative abundance of AT2 cells, these exploratory analyses suggest that nerandomilast is associated with altered alveolar epithelial cell‐state dynamics originating from AT2 cells during the repair phase following lung injury.

### 2.4 Nerandomilast is Associated With Preservation of Alveolar Epithelial Architecture Adjacent to Fibrotic Lesions

Given the transcriptional changes identified by scRNA‐seq, we examined whether these findings were reflected in alveolar epithelial architecture in vivo. H&E staining and immunohistochemical analysis of SFTPC, a major marker of AT2 cells, revealed a greater number of hypertrophic SFTPC‐positive AT2 cells (red arrows) in the nerandomilast‐treated group than in the control group on Day 14 (*p* = 0.0185; Figure 4A,B). The SFTPC‐positive AT2 cells were aligned in a single layer adjacent to the fibrotic lesions, and the alveolar architecture was preserved in the nerandomilast‐treated group compared to the controls, with fewer collapsed alveolar regions. Consistent with these findings, flow cytometry analysis demonstrated an increased relative abundance of total epithelial and AT2 cells in the nerandomilast‐treated group on Day 14 (epithelial cells: *p* = 0.0019; AT2 cells: *p* = 0.0190; Figures 4C and S2B). Next we evaluated epithelial cell cycle activity and transitional epithelial states using Ki67 and KRT8 immunostaining. The KRT8‐positive area and proportion of Ki67‐positive alveolar epithelial cells were significantly increased in the nerandomilast‐treated group on Day 14 (KRT8, *p* = 0.0025; Ki67, *p* = 0.0025; Figure 4A,B). Notably, multiple Ki67‐positive epithelial cells were also aligned in a single layer near the fibrotic area following nerandomilast treatment, whereas Ki67‐positive cells in control lungs were observed as isolated single cells within fibrotic regions (Figure 4A). Double immunofluorescence staining for SFTPC and PDPN was performed to further evaluate the spatial organization of the alveolar epithelium (Figure 4D). In the nerandomilast‐treated group, SFTPC‐positive AT2 cells were more frequently observed within and adjacent to cell‐dense lesions and PDPN‐positive AT1 cells formed a more continuous alveolar epithelial lining. In contrast, the control group had fewer SFTPC‐positive AT2 cells and a more fragmented distribution of PDPN‐positive AT1 cells in the lesions. These observations further support the preservation of alveolar epithelial architecture following nerandomilast treatment.

**FIGURE 4:**
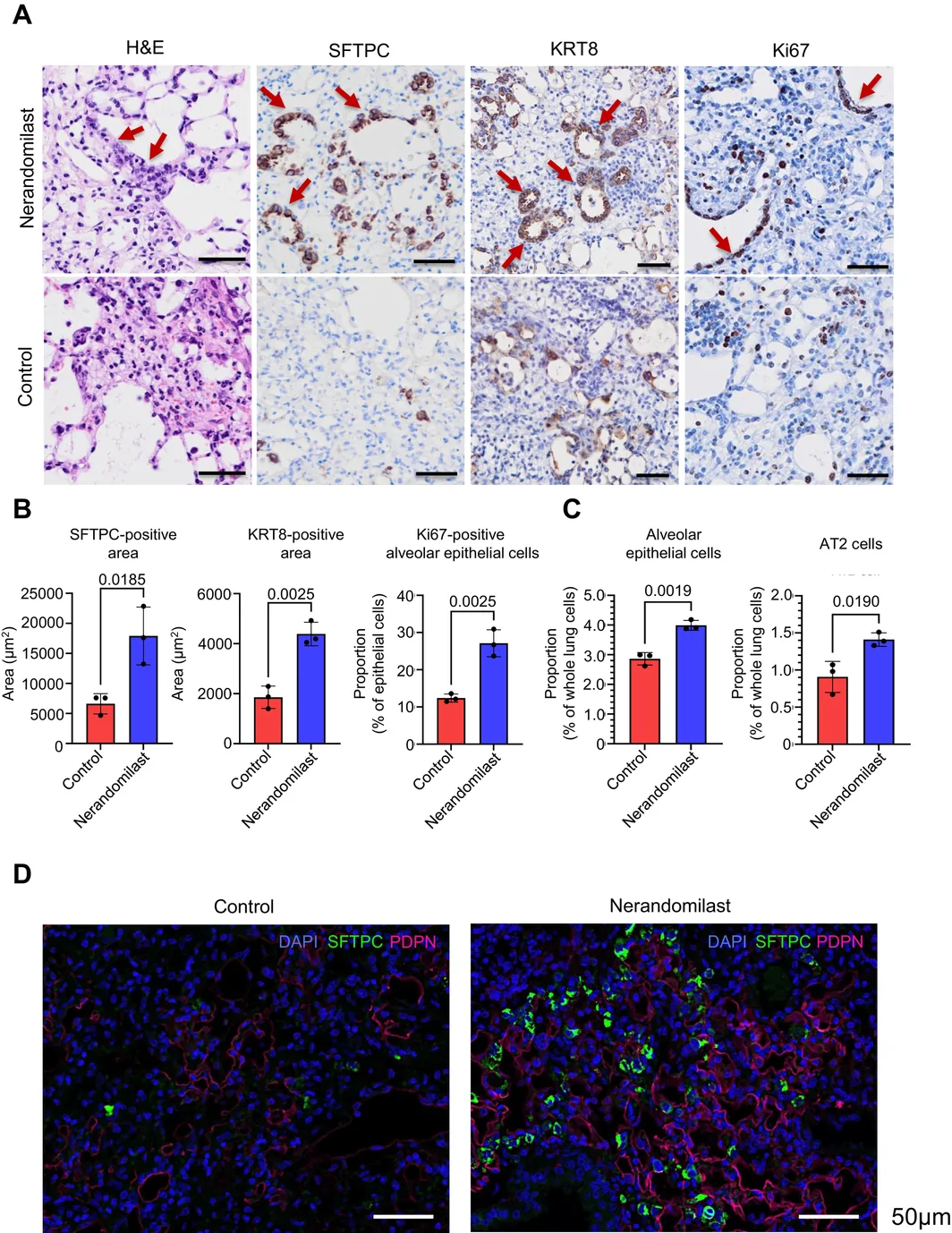
Nerandomilast preserves alveolar epithelial architecture adjacent to fibrotic lesions. (A) Representative H&E and immunostaining for SFTPC, KRT8, and Ki67 in lungs from control and nerandomilast‐treated mice collected on Day 14. Scale bar: 50 μm. (B) Quantification of SFTPC‐positive area, KRT8‐positive area, and the proportion of Ki67‐positive alveolar epithelial cells on Day 14. *p* < 0.05 by student's *t*‐test. Each point represents one mouse (*n* = 3 mice per group). (C) Flow cytometry analysis of alveolar epithelial cell (EpCAM^+^) and AT2 cell (EpCAM^+^, IA/IE^+^) populations in the whole lung collected on Day 14. Each point represents one mouse (*n* = 3 mice per group). (D) Representative double‐immunofluorescence images of SFTPC (AT2 cells, green) and PDPN (AT1 cells, magenta) in control and nerandomilast‐treated mice on Day 14 after BLM administration. Scale bars, 50 μm.

Taken together, these findings suggest that nerandomilast treatment preserves alveolar epithelial architecture and is associated with altered AT2 cell dynamics during lung repair.

## 3 Discussion

In this study, we investigated the cellular responses associated with nerandomilast administration in a BLM‐induced mouse model of pulmonary fibrosis using histological and single‐cell transcriptomic analyses. The results revealed that nerandomilast, a preferential PDE4B inhibitor, attenuated fibrotic remodeling while preserving the structure of the alveolar epithelium. These changes were accompanied by an increased relative abundance of AT2 cells, accelerated directional flow from AT2 cells toward the CLDN4^+^ state, and activation of cell cycle‐related transcriptional pathways in AT2 cells. These findings suggest that modulation of alveolar epithelial dynamics is a key cellular response to nerandomilast treatment.

Recent studies on nerandomilast have clarified its effects on inflammation, the mesenchymal system, and the vascular system. Nerandomilast has been shown to inhibit skin and lung fibrosis in a BLM‐induced mouse model of systemic sclerosis‐associated interstitial lung disease (Liu et al. 2025). It also induces dedifferentiation of myofibroblasts in human idiopathic pulmonary fibrosis and diminishes their contractility in vitro (Reininger et al. 2025). Furthermore, it has been shown to mitigate vascular dysfunction by strengthening the endothelial junctions (Reininger et al. 2025). In contrast, the effects of nerandomilast on the alveolar epithelium remain poorly understood. Our findings extend these previous observations by providing new evidence that nerandomilast modulates the alveolar epithelial response.

The alveolar epithelium is composed of AT1 cells, which mediate gas exchange, and AT2 cells, which secrete surfactants and serve as resident stem cells (Barkauskas et al. 2013). Following epithelial injury, AT2 cells contribute to epithelial regeneration via self‐renewal and differentiation into AT1 cells (Barkauskas et al. 2013). The detection of PDE4B in alveolar regions, including in cells with morphological features consistent with those of AT2 cells, together with the preservation of alveolar epithelial architecture in nerandomilast‐treated mice after bleomycin challenge, raises the possibility that PDE4B inhibition contributes to the modulation of alveolar epithelial cell state dynamics, in addition to its previously reported immunomodulatory and anti‐fibrotic effects. Previous studies demonstrated that PDE4 inhibition attenuates lung fibrosis in models of AT2 cell‐selective injury‐induced pulmonary fibrosis and reduces the expression of the epithelial injury marker SP‐D (Sisson et al. 2018). In addition, pan‐PDE inhibition has been shown to directly modulate the response of alveolar epithelial cells to fibrogenic stimuli and suppress TGFβ‐induced epithelial‐mesenchymal transition (Wójcik‐Pszczoła et al. 2022). These findings support the possibility that PDE4B inhibition influences alveolar epithelial responses. Therefore, the changes observed in alveolar epithelial cells following nerandomilast administration may contribute to the overall tissue repair process.

Interestingly, nerandomilast treatment induced profound changes in AT2 cell population. Flow cytometric analysis and scRNA‐seq showed an increased relative abundance of AT2 cells on Day 14 following nerandomilast treatment. Furthermore, differential expression analysis revealed that several genes upregulated in AT2 cells following nerandomilast treatment. Among the cell cycle‐associated genes, Ube2c and Birc5 remained significantly upregulated after false discovery rate (FDR) correction, whereas Mki67, Top2a, Ccna2, and Cdk1 showed consistent upward trends but did not reach statistical significance after FDR correction (Table S1). Pathway analysis demonstrated increased cell cycle‐associated transcriptional activity in AT2 cells. PDE4 inhibition increases intracellular cAMP levels of expression and activates multiple downstream transcriptional programs, including those mediated by CREB (Blauvelt et al. 2023; Aringer et al. 2024). Therefore, cAMP‐dependent signaling may contribute to the cell‐cycle‐related transcriptional profile observed in AT2 cells. Consistent with this possibility, activation of the cAMP–PKA–CREB pathway has been reported to promote AT2 cell survival under oxidative stress (Barlow et al. 2008). In addition, nerandomilast has been shown to increase CREB phosphorylation and activate cAMP‐dependent signaling pathways in experimental models of hypersensitivity pneumonitis and myositis‐associated interstitial lung disease (Shi et al. 2026; Cui et al. 2026). Taken together, these findings suggest that nerandomilast treatment can contribute to an increased relative abundance of AT2 cells and enhancement of cell‐cycle‐associated transcriptional activity in AT2 cells observed in the present study.

Furthermore, our scRNA‐seq analysis identified two distinct clusters of epithelial transitional cells, in addition to AT1 and AT2 cells, characterized by a high expression of CLDN4 and CLDN18. These populations closely resemble the recently proposed pre‐alveolar type‐1 transitional states (PATS), termed PATS‐1 and PATS‐2 (Kobayashi et al. 2020; Konkimalla et al. 2022). Consistent with previous reports, PATS‐1, marked by *Cldn4* and *Sfn*, represents a transitional state associated with stress and regeneration (Kobayashi et al. 2020). In contrast, PATS‐2, which is enriched in *Cldn18*, *Hopx*, and *Ager*, corresponds to a more advanced state committed to AT1 differentiation (Kobayashi et al. 2020). Consistent with these reports, the CLDN4^+^ cluster in our dataset exhibited transcriptional features similar to those of PATS‐1, whereas the CLDN18^+^ cluster in our dataset resembled those of PATS‐2, showing increased expression of AT1 lineage markers. Moreover, RNA velocity‐derived pseudotime analysis using our dataset revealed a stepwise trajectory starting from AT2 cells, passing through our CLDN4^+^ cells and then our CLDN18^+^ cells, and finally reaching AT1 cells. Previous studies identified KRT8‐positive transitional epithelial cells as an intermediate state during alveolar regeneration. However, the persistent accumulation of these cells has also been associated with maladaptive epithelial remodeling in fibrotic lungs (Strunz et al. 2020). In the present study, an increase in KRT8‐positive cells occurred together with an increased relative abundance of AT2 cells, preservation of alveolar epithelial architecture, and attenuation of fibrosis. In addition, velocity analysis suggested increased directional flow from AT2 cells toward the CLDN4^+^ state. Collectively, these findings support the interpretation that PDE4B inhibition alters alveolar epithelial‐cell‐state dynamics during alveolar repair.

The mechanism of action of nerandomilast elucidated in this study is highly significant for IPF, a disease in which AT2 cell dysfunction is considered a central pathogenic driver. Previous studies have consistently reported AT2 cell hyperplasia, apoptosis, and aberrant epithelial remodeling in the lungs of patients with IPF (Parimon et al. 2020; Zhu et al. 2022; Confalonieri et al. 2022). In addition, mutations in AT2 cell–specific genes, including those that encode surfactant‐associated proteins, are strongly associated with familial pulmonary fibrosis (Chibbar et al. 2004; Wang et al. 2009). These observations highlight the critical role of AT2 cells in maintaining alveolar homeostasis and repairing the injured epithelium. Therefore, our findings suggest a previously underappreciated role of PDE4B inhibition in altering alveolar epithelial cell‐state dynamics and provide a potential mechanistic link between epithelial repair and the anti‐fibrotic efficacy of nerandomilast.

Nevertheless, this study has several limitations. First, the number of biological samples used for scRNA‐seq was limited, and the changes in AT2 and transitional epithelial cells identified in this study require validation in a larger cohort. Secondly, we evaluated the effects of nerandomilast at a relatively early stage of fibrosis using a single‐dose BLM model. Therefore, it remains unclear whether similar effects are observed in more severe fibrosis or in chronic conditions. Future studies will require validation using repeated‐dose BLM models or long‐term observation systems. Third, although this study demonstrated an increased relative abundance of AT2 cell and cell cycle‐related pathways following nerandomilast administration, the molecular mechanisms by which PDE4B inhibition regulates alveolar epithelial cell state dynamics remain unclear. Detailed mechanistic analyses, including cAMP signaling and its downstream pathways, are required in the future.

In conclusion, our findings provide new evidence that nerandomilast is associated with an altered alveolar epithelial response in experimental pulmonary fibrosis. Nerandomilast treatment was associated with an increased relative abundance of AT2 cells and increased cell cycle‐associated transcriptional activity of AT2 cells. These changes were accompanied by the preservation of the alveolar epithelial architecture during lung injury and repair. Collectively, our findings suggest that modulation of alveolar epithelial responses may represent an additional cellular effect of PDE4B inhibition, providing new mechanistic insights into the anti‐fibrotic effects of nerandomilast.

## 4 Experimental Procedures

### 4.1 Compound

Nerandomilast was synthesized at the chemical facilities of Boehringer Ingelheim (Biberach, Germany), as described in patent WO 2013/026797 A1. Nerandomilast was administered orally at a dose of 12.5 mg/kg twice daily, starting on Day 8 after bleomycin instillation and continued until the indicated endpoints (Days 10 and 14), in accordance with previously reported data from Boehringer Ingelheim, Germany. The control group consisted of vehicle‐treated mice that received the same volume of vehicle via the same oral gavage route and dosing schedule as the mice treated with nerandomilast. The vehicle solution for oral gavage consisted of 0.5% hydroxyethyl cellulose 200–300 mPa s, 2% in water at 20°C (H0242; Tokyo Chemical Industry Co. Ltd., Tokyo, Japan) with 0.01% Tween‐20 (35624‐15; Nacalai Tesque, Kyoto, Japan) in water. Briefly, 1 g of hydroxyethyl cellulose was dispersed in 200 mL of drinking water and allowed to swell for 30 min, followed by stirring at room temperature until complete dissolution. Tween‐20 was added at a final concentration of 0.01%. The vehicle was stored at 4°C and used within 2 weeks.

### 4.2 Laboratory Animals

Young male C57BL/6J mice (8–10 weeks old; CLEA Japan, Tokyo, Japan) were used to generate a pulmonary fibrosis model. All mice were housed in a specific pathogen‐free facility under a standard 12‐h light/dark cycle with ad libitum access to food and water. Bleomycin (BLM, 2 mg/kg body weight; Nippon Kayaku, Tokyo, Japan) was intratracheally instilled under anesthesia. Oral nerandomilast (12.5 mg/kg) or vehicle was administered twice daily beginning on Day 8 after BLM injection. The treated mice were euthanized under deep isoflurane anesthesia on Days 0, 8, 10, and 14 after the BLM injection for sample collection. Tissue samples were collected for further analysis. The body weights of the mice were recorded daily, and animals showing > 20% weight loss or severe distress were humanely euthanized (*n* = 3–7 mice per group per time point). Blinding was not performed because the treatment allocation could be inferred from the experimental procedures and outcomes.

### 4.3 Measurement of Hydroxyproline in the Mouse Lung

To estimate the amount of collagen in the lungs, the right lung was subjected to a hydroxyproline assay (QZBhypro1; QuickZyme Biosciences) according to the manufacturer's protocol.

### 4.4 Histopathology

Whole lung lobes were fixed in 4% paraformaldehyde (PFA) in PBS for 24 h and embedded in paraffin. Sections (3 μm thick) were cut using a paraffin microtome with a stainless‐steel knife, mounted on glass slides, deparaffinized with xylene, dehydrated through a graded ethanol series, and stained with hematoxylin and eosin. Trichrome staining was performed according to the manufacturer's protocol (ab150686; Abcam) to assess collagen deposition. Morphological analysis of the fibrotic areas was performed by quantifying the trichrome‐positive areas using the Image Joint System. For each mouse, three image fields from one tissue section were analyzed, and the quantified values from the three fields were averaged to obtain a single representative value for each animal, which was used for statistical analysis.

### 4.5 Immunohistochemistry (IHC) of Tissue Sections

Formalin‐fixed, paraffin‐embedded 3‐μm‐thick sections of the whole lung lobes were stained using standard procedures. Briefly, the sections were deparaffinized using a graded series of alcohols and washed with PBS. Heat‐activated antigen retrieval was performed for 30 min in a heating chamber using a target retrieval solution (S1699; DAKO for SFTPC and Ki67, 415201; NICHIREI BIOSCIENCES for KRT8). Endogenous peroxidases were blocked for 30 min at room temperature using the Dako REAL Peroxidase‐Blocking Solution (S2023; DAKO, Glostrup, Denmark). The slides were blocked for nonspecific protein binding by incubation for 30 min at room temperature in Blocking One Histo (06349‐64; Nacalai Tesque).

Tissue was immunostained with rabbit anti‐mouse SFTPC antibody (ab211326; Abcam, 1:2000 dilution), rabbit anti‐mouse Ki67 antibody (NB110‐89719; Novus Biologicals, 1:500 dilution), or rabbit anti‐mouse KRT8 antibody (ab53280; Abcam, 1:300 dilution) diluted with Dako REAL Antibody Diluent (S2022; DAKO) overnight at 4°C. A horseradish peroxidase‐conjugated secondary antibody (8114S; Cell Signaling Technology, Danvers, MA, USA) was applied to the sections for 60 min at room temperature. Positive reactions were visualized using a 3,3′‐diaminobenzidine (DAB) substrate kit (K3468; DAKO). Sections were lightly counterstained with hematoxylin.

For quantitative analysis, three image fields from one tissue section were analyzed for each mouse. SFTPC‐ or KRT8‐positive areas were quantified using the Image Joint System, and Ki67 staining was quantified as the proportion of Ki67‐positive alveolar epithelial cells. The measurements obtained from the three fields were averaged to generate a single representative value for each animal, and these animal‐level values were used for statistical analysis.

Double immunofluorescence staining for SFTPC and PDPN was performed on the lung sections. Sections were incubated with rabbit anti‐mouse SFTPC antibody (ab211326; Abcam; 1:2000) and Syrian hamster anti‐mouse PDPN antibody (ab11936; Abcam; 1:400) overnight at 4°C. Donkey anti‐rabbit IgG (H + L) secondary antibody conjugated to Rhodamine Red‐X (711‐296‐152; Jackson ImmunoResearch) for SFTPC, and goat anti‐Syrian hamster IgG (H+L) cross‐absorbed secondary antibody conjugated to Alexa Fluor 647 (A78902; Invitrogen) for PDPN were applied to the sections for 60 min at room temperature. Nuclei were counterstained with DAPI. Whole‐slide fluorescence images were acquired using an Olympus VS200 Research Slide Scanner (Olympus) and visualized using Olyvia software. The spatial distribution of SFTPC‐positive AT2 and PDPN‐positive AT1 cells within and adjacent to lesion areas was qualitatively evaluated.

### 4.6 Fluorescence‐Activated Cell Sorting (FACS)

Lungs were excised from the mice. Primary lung cells were dissociated from lung tissue using collagenase type 1 (LS004196; Worthington, 450 U/mL), dispase (354235; Corning, Corning, NY, USA, 5 U/mL), and DNase I (1003347370; Sigma‐Aldrich, St. Louis, MO, USA, 2 KU/mL) in RPMI 1640 medium (30264‐56; Nakalai Tesque) using a gentleMACS Octo Dissociator with Heaters (Miltenyi Biotec, Bergisch Gladbach, Germany) for 30 min at room temperature. The resulting suspension was filtered through a 40 μm cell strainer, centrifuged at 400 × *g* for 3 min, and treated with ACK lysis buffer (A10492‐01; Gibco) for 3 min. Before antibody staining, Fc receptors were blocked using an anti‐mouse CD16/CD32 antibody (553141; BD Biosciences, Franklin Lakes, NJ, USA) at 1 μg per 10^6^ cells for 10 min at 4°C. Cells were stained with anti‐mouse CD45‐Brilliant Violet 41 (103134; BioLegend, San Diego, CA, USA, 1:200), CD31‐APC (102410; BioLegend, 1:200), CD140a‐PE (135906; BioLegend, 1:200), CD326‐PerCP (118220; BioLegend, 2:100), and IA/IE‐FITC (107606; BioLegend, 1:100). Single‐stained samples were used for compensation, and unstained controls were included to determine the background fluorescence and assist in setting the positive gates. Gating was performed sequentially by excluding debris and doublets (FSC‐A vs. FSC‐H) and subsequently identifying lung CD45^−^ CD31^−^ CD140a^−^ CD326^+^ epithelial cells. AT2 cells were defined as lung CD45^−^ CD31^−^ CD140a^−^ CD326^+^ and IA/IE^+^ cells. On average, 1 × 10^5^ events were recorded per sample, and three biological replicates were analyzed per group. The principal gating strategy, including the subsequent identification of lung epithelial cells and AT2 cells, is shown in Figure S2C.

### 4.7 qRT‐PCR

Total RNA was isolated from the lung tissues using a High Pure RNA Isolation Kit (11828665001; Roche) according to the manufacturer's instructions. The quantity and purity of RNA were assessed using a NanoDrop 1000 spectrophotometer by measuring the A260/A280 ratio. Reverse transcription was performed using the ReverTra Ace qPCR RT Master Mix (Toyobo, Osaka, Japan). Quantitative reverse‐transcription PCR (qRT‐PCR) was conducted using the TaqMan Gene Expression Assay (Thermo Fisher Scientific, Waltham, MA, USA) for *Tgfb1*, *Col1a1*, and *18S*. Relative mRNA expression levels were analyzed using the comparative Ct (ΔΔCt) method, with normalization to 18S rRNA (Livak and Schmittgen 2001).

### 4.8 Quantification and Statistical Analysis

All quantitative data are presented as the mean ± standard deviation (SD). Differences between the two groups were analyzed using the student's *t*‐test, except for body weight data on Day 14, which were analyzed using a two‐tailed Mann–Whitney *U* test. Multiple groups were compared using one‐way analysis of variance (ANOVA) followed by Tukey's correction. Statistical significance was set at *p* < 0.05.

### 4.9 scRNA‐seq

Lungs were excised from the mice. Primary lung cells were dissociated from the lung tissue using collagenase type 1 (LS004196; Worthington, 450 U/mL), dispase (354235; Corning, 5 U/mL), and DNase (1003347370; Sigma‐Aldrich, 2 KU/mL) in RPMI 1640 medium (30264‐56; Nakalai Tesque) using a gentleMACS Octo Dissociator with Heaters (Miltenyi Biotec) for 30 min at room temperature. The cells were centrifuged at 400 × *g* for 3 min and treated with ACK lysis buffer (A10492‐01; Gibco) for 3 min. The lung cell pellet was resuspended in MACS buffer (1× PBS, pH 7.2, 0.5% BSA, and 2 mM EDTA). Dead cells were magnetically removed using QuadroMACS separators, LS columns, and a Dead Cell Removal Kit (130‐090‐101; Miltenyi Biotec). Live cells were resuspended in 0.04% BSA in PBS. Cell viability was evaluated by trypan blue staining, and only samples exhibiting viability greater than 80% (1 × 10^3^ cells/μL in 100 μL per sample) were submitted for single‐cell RNA sequencing (scRNA‐seq).

Single‐cell suspensions were processed using a 10× genomics chromium controller to generate single‐cell gel beads‐in‐emulsions (GEMs) targeting 20,000 cells per sample. Following reverse transcription, cDNA amplification, and library construction according to the chromium single cell 5′ v3 (GEM‐X) protocol, library quality was validated using an Agilent Bioanalyzer. Libraries were sequenced on an Illumina NovaSeq X Plus sequencer with a minimum depth of 200 million reads per sample.

### 4.10 scRNA‐seq Data Preprocessing and Quality Control

Raw sequencing data were processed using Cell Ranger software (version 8.0.1, 10× Genomics, Pleasanton, CA, USA). Reads were aligned to the mouse genome reference refdata‐gex‐GRCm39‐2024‐A. The expression data were processed using the Python package Scanpy (Wolf et al. 2018). The initial filtering removed cells with fewer than 100 detected genes. Genes detected in fewer than three cells were excluded from analysis. Cells with mitochondrial transcript percentages exceeding 20% were excluded from analysis. Gene counts were log‐transformed and normalized using the Scanpy function.

Data from individual samples were integrated using the Harmony (Korsunsky et al. 2019) batch correction algorithm, as implemented in Scanpy. Dimensionality reduction was performed using a principal component analysis (PCA). The cells were visualized using UMAP and clustering was performed using the Leiden algorithm. Cell clusters were manually inferred based on marker gene expression patterns and annotated by re‐labeling using Bio Turing BBrowserX (Le et al. 2020).

### 4.11 Differential Expression Analysis

Differential expression analysis was performed using the pseudobulk approach (Squair et al. 2021). Following the selection of target cells, counts were aggregated across cells for each biological replicate to generate pseudo‐bulk data. The differentially expressed genes were identified using PyDESeq2. For downstream analyses, genes were ranked based on the Wald test statistic (Stat) calculated using DESeq2, and the top‐ranked genes were selected for further evaluation. Differential gene expression analysis of the CLDN4^+^ and CLDN18^+^ transitional epithelial cell populations was performed using the Venice algorithm implemented in Browser X (Bioturing, San Diego, CA, USA). The results were visualized as volcano plots, with log2 fold change (log2FC) on the *x*‐axis and −log10 FDR on the *y*‐axis.

### 4.12 Pathway Enrichment Analysis

Pathway enrichment analysis was performed using the Enrichr platform (Chen et al. 2013). KEGG pathway enrichment analysis was performed using the top 100 upregulated and downregulated genes ranked by the Wald statistic (Stat) obtained from the differential expression analysis (Kanehisa and Goto 2000). This approach was used to identify biological pathways represented by the most strongly altered transcriptional signatures while maintaining a consistent number of input genes across comparisons. Pathway enrichment significance was evaluated using Benjamini–Hochberg‐adjusted *p* values.

### 4.13 RNA Velocity Analysis

Spliced and unspliced gene counts were determined using Velocyto (La Manno et al. 2018) followed by further analysis using scVelo (Bergen et al. 2020). RNA velocities were calculated using the standard scVelo pipeline and projected onto UMAP embedding. RNA velocity‐inferred pseudotime trajectory analysis was subsequently performed using scVelo with AT2 cells, defined as the root state. The analysis was repeated for all biological replicates and yielded highly consistent trajectory structures across samples. A representative trajectory from Day 14 in the nerandomilast‐treated group is shown in the main figure.

### 4.14 Quantification of Velocity Trend

To quantify the velocity trend from AT2 cells to the transition states, we defined a specific region of interest (ROI) (indicated by the red ellipse in Figure 3E). The ROI was manually defined on the integrated UMAP embedding as an intermediate area between the annotated AT2 and CLDN4^+^ clusters. The reference direction was defined as the vector connecting AT2 and CLDN4^+^ centroids. The transition strength was quantified as the average projection of RNA velocity vectors onto this reference direction within the ROI. The same ROI boundaries and reference directions were applied consistently to all samples.

## Author Contributions

**Nanako Hamada:** writing – original draft, investigation, formal analysis, visualization, methodology, validation. **Yoshiaki Hayashi:** investigation, formal analysis, visualization, validation, software. **Daisuke Motooka:** data curation, supervision, writing – review and editing. **Atsushi Kuwahara:** investigation, data curation. **Shizuo Akira:** supervision, resources. **Kiyoharu Fukushima:** funding acquisition, project administration, methodology, conceptualization.

## Funding

This work was supported by the Boehringer Ingelheim.

## Ethics Statement

All mouse experiments were reviewed and approved by the Animal Care and Use Committee at the World Premier Institute Immunology Frontier Research Center, Osaka University.

## Conflicts of Interest

The authors meet the criteria for authorship as recommended by the International Committee of Medical Journal Editors. This study was supported by Boehringer Ingelheim. Boehringer Ingelheim had no role in the design, analysis, or interpretation of the results in this study. Boehringer Ingelheim was given the opportunity to review the manuscript for medical and scientific accuracy as it relates to Boehringer Ingelheim substances, as well as intellectual property considerations.

## Supporting information


**Figure S1:** PDE4B expression in other lung cell types. (A) Representative immunostaining images showing PDE4B expression in lung sections from BLM‐induced mice (Days 0, 10, and 14). PDE4B expression was examined in vascular endothelial cells (red arrows), smooth muscle cells (blue arrows), and bronchial epithelial cells (outlined with dashed lines) (*n* = 3 mice per group). (B) PDE4B expression in neutrophils (red arrows) and lymphocytes in mouse lungs. PDE4B expression was undetectable in vascular endothelial cells, neutrophils, and lymphocytes. Smooth muscle cells showed weak positivity only in mice, whereas bronchial epithelial cells displayed weak staining in BLM administrated mice but were negative in healthy controls. Scale bars: 50 μm.
**Figure S2:** Gating strategy and marker genes used for cell type annotation. UMAP plots showing the representative expression of EPCAM, CD68, and PECAM used for annotation. A list of marker genes applied for annotation is provided. (B) Relative abundance of alveolar epithelial cell populations (AT1, AT2, CLDN4^+^, and CLDN18^+^) on Day 10 (*n* = 2 biological replicates per group). (C) Representative flow cytometry gating strategy for the isolation of alveolar epithelial cells and AT2 cells.

**Table S1:** Top 100 upregulated and downregulated genes ranked by Wald statistic. Genes were ranked by the Wald statistic (stat), and the top 100 upregulated and top 100 downregulated genes were selected from the comparison between control and nerandomilast‐treated mice.

## Data Availability

The raw sequencing data of this manuscript has been uploaded to the GEO database under the accession number GSE306008. The source data are provided in this study. The custom code for reproducing the results is available at https://github.com/yoshiF7d/nerandomilast.
